# Supplementary material for: Selective regulation of transsynaptic alignment and postsynaptic assembly by a novel NCAM family synaptic adhesion molecule
Source: bioRxiv. 2026 Mar 16:2026.03.13.710942. Preprint. [Version 1] doi: 10.64898/2026.03.13.710942 (PMC13015403; doi:10.64898/2026.03.13.710942)
Supplement: Supplement 1 [file NIHPP2026.03.13.710942v1-supplement-1.pdf]

bioRxiv preprint doi: https://doi.org/10.64898/2026.03.13.710942; this version posted March 16, 2026. The copyright holder for this preprint (which was not certified by peer review) is the author/funder, who has granted bioRxiv a license to display the preprint in perpetuity. It is made available under aCC-BY 4.0 International license.

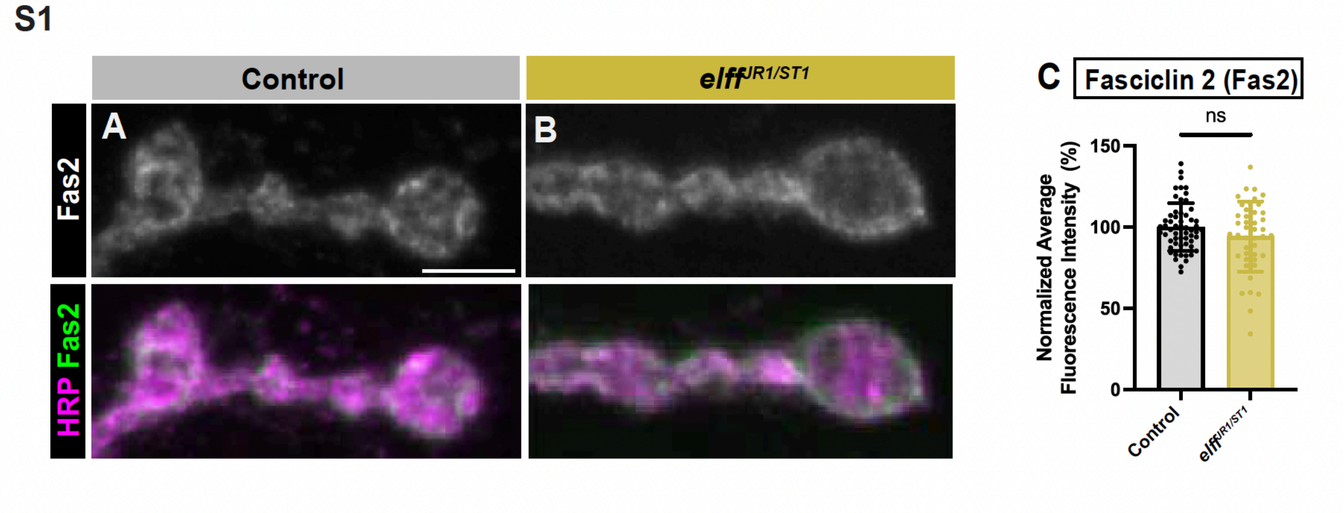

Supplemental Figure S1.

(A-B) Z-projections of NMJ boutons of the indicated genotypes stained for Fas2 (green) and HRP (magenta). (C) Quantification of Fas2 fluorescence intensity. Data are mean values normalized to control ± SD (control: 100.0 ± 14.71, *elff*$^{JR1/ST1}$: 94.19 ± 21.59). Significance determined by unpaired t-test [ns, not significant]. n ≥ 43, Animals ≥ 11.

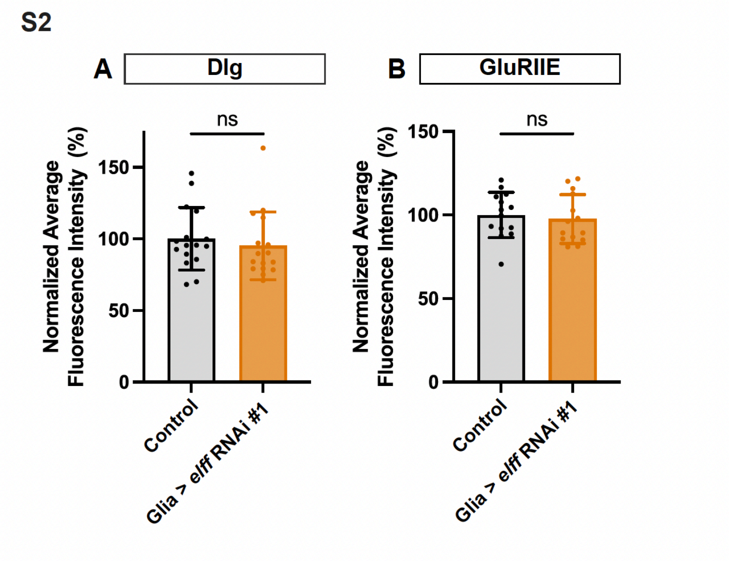

Supplemental Figure S2.

(A-B) Quantification of fluorescence intensity of Dlg (A) and GluRIIE (B) staining in third instar larval NMJs at muscle 4 from the indicated genotypes. The Gal4 driver used for glia is RepoGal4. Data are mean values normalized to control ± SD for both Dlg (control: 100.0 ± 21.78, glia > *elff* RNAi: 95.15 ± 23.68) and GluRIIE (control: 100.0 ± 13.60, glia > *elff* RNAi: 97.54 ± 14.62). Significance determined by Mann-Whitney test (A) or unpaired t-test (B) [ns, not significant]. n ≥ 14, Animals ≥ 4.
